# Risk Factors for Breast Cancer Among Chinese Women: A 10-Year Nationwide Multicenter Cross-Sectional Study

**DOI:** 10.2188/jea.JE20120217

**Published:** 2014-01-05

**Authors:** Hui Lee, Jia-Yuan Li, Jin-Hu Fan, Jing Li, Rong Huang, Bao-Ning Zhang, Bin Zhang, Hong-Jian Yang, Xiao-Ming Xie, Zhong-Hua Tang, Hui Li, Jian-Jun He, Qiong Wang, Yuan Huang, You-Lin Qiao, Yi Pang

**Affiliations:** 1 West China School of Public Health, Sichuan University, Sichuan, China; 2 Department of Cancer Epidemiology, Cancer Institute & Hospital, Chinese Academy of Medical Sciences & Peking Union Medical College, Beijing, China; 3 Center of Breast Disease, Cancer Institute & Hospital, Chinese Academy of Medical Sciences & Peking Union Medical College, Beijing, China; 4 Department of Breast Surgery, Liaoning Cancer Hospital, Shenyang, China; 5 Department of Breast Surgery, Zhejiang Cancer Hospital, Hangzhou, China; 6 Department of Breast Oncology, Sun Yat-Sen University Cancer Center, Gungzhou, China; 7 Department of Breast-Thyroid Surgery, Xiangya Second Hospital, Central South University, China; 8 Department of Breast Surgery, Second People’s Hospital of Sichuan Province, Chengdu, China; 9 Department of Oncosurgery, First Affiliated Hospital of Medical College, Xi’an Jiao Tong University, Xi’an, China

**Keywords:** breast cancer, risk factors, nationwide, multicenter

## Abstract

**Background:**

The characteristics of established risk factors for breast cancer may vary among countries. A better understanding of local characteristics of risk factors may help in devising effective prevention strategies for breast cancer.

**Methods:**

Information on exposures to risk factors was collected from the medical charts of 4211 women with breast cancer diagnosed during 1999–2008. The distributions of these exposures among regions, and by menopausal status and birth period, were compared with the χ^2^ test. Crude associations between the selected factors and breast cancer were estimated using the cases in the present study and a representative control population, which was selected from qualified published studies.

**Results:**

As compared with cases from less developed regions, those from more developed regions were significantly more likely to be nulliparous, had fewer childbirths (*P* < 0.05), and were less likely to have breastfed (*P* = 0.08). As compared with premenopausal cases, postmenopausal cases were more likely to be overweight and to have breastfed and had more childbirths (*P* < 0.05). The number of live births and rate of breastfeeding decreased in relation to birth period (*P* for trends <0.001). Overweight, late menopause, and family history of breast cancer were significantly associated with breast cancer among Chinese women.

**Conclusions:**

Breast cancer incidence was associated with nulliparity and history of breastfeeding. Population attributable risks should be assessed, especially for more developed areas and young women. The effects of body mass index, age at menopause, and family history of breast cancer should be given priority during assessment of breast cancer risk among Chinese women.

## INTRODUCTION

According to global cancer statistics for the year 2008, breast cancer was the most frequently diagnosed malignancy among women worldwide (incidence, approximately 1.38 million per year) and resulted in 46 million deaths.^1^ The causes of breast cancer are not well understood. However, a number of risk factors for breast cancer are supported by strong evidence, especially in Western countries. Population-based studies have shown that reproductive factors, including early menarche, late menopause, nulliparity, and absence of a history of breastfeeding, increase the risk of breast cancer.^2^ Several lifestyle-related risk factors have also been shown to contribute to breast cancer development, including lack of physical activity, overweight, smoking, alcohol drinking, oral contraceptive use, hormone replacement therapy, poor dietary intake, and radiation exposure.^3^ In addition, studies of families with high breast cancer incidence have shown that about 5% to 7% of all breast carcinomas are hereditary.^4^

Although the mechanisms underlying breast cancer risk require further clarification, experience in Western countries has highlighted the benefits of awareness and understanding of modifiable factors. The publication of the Women’s Health Initiative randomized trial in the United States led to a rapid decrease in the number of women receiving postmenopausal women hormone replacement, which might partly explain the sharp decline in breast cancer incidence in the United States and other Western countries.^5^ In addition, using nationally representative information on risk factors for breast cancer, a method of identifying women at high risk for breast cancer (the Gail model) has been used in clinical counseling and breast cancer prevention studies in North America and Europe.^6^

Although Chinese women are at relatively low risk for breast cancer, age-standardized breast cancer incidence and mortality rates have risen rapidly in China during the past 2 to 3 decades, making it the leading cancer among women.^7^ Average age at breast cancer onset is approximately 10 years earlier among Chinese women as compared with Western women. Thus, the disease burden in China has substantially increased. Because most established risk factors are related to lifestyle, which is influenced by culture and economic development, disparities may exist between China and Western countries in population distribution and the effects of risk factors. To date, primary prevention has received little attention in China, partly because of insufficient knowledge of risk factors for breast cancer among Chinese women. In relation to secondary prevention, it has been suggested that implementing mammography screening programs in populations at high risk for breast cancer would be more cost-effective in low-income countries.^8^ Therefore understanding the characteristics of risk factors in China might help identify high-risk groups and improve secondary prevention.

Currently, there are few national monitoring data on risk factors among the Chinese general population. High-quality epidemiologic studies of breast cancer risk factors in China have predominately been conducted in regions with greater development and higher economic status, and the results may thus not represent the entire country. In this 10-year (1999–2008) nationwide multicenter retrospective clinical epidemiologic study of female breast cancer in China, we examined the distribution of risk factors among Chinese women with breast cancer. Because exposure to most risk factors in a population is influenced by local culture, policy, and economic status, disparities in distributions among cancer cases may indicate possible differences in distribution in their source population. In addition, we estimated associations between selected risk factors and breast cancer by retrospectively reviewing representative national data on cases and comparing those findings with information on controls reported in previous studies. We hope our results provide clues for further study of the Chinese population and contribute to the development of appropriate prevention strategies in China.

## METHODS

### Study design and data collection

We conducted a 10-year (1999–2008) nationwide multicenter retrospective clinical epidemiology study of female breast cancer in China. The study protocols were approved by the Institutional Review Board of the Cancer Foundation of China.

Hospital selection and case sampling methods have been previously described.^7^ In brief, to ensure that our sample was geographically representative of China, 1 tertiary hospital was selected in each of 7 geographic regions of China, ie, north, northeast, northwest, central, east, south, and southwest. These regions encompass most of the country and represent different levels of breast cancer burden. We selected leading regional public cancer hospitals and referral centers that provide pathologic diagnosis, surgery, radiotherapy, medical oncology, and routine follow-up care for women with breast cancer. Patients visiting the selected hospitals were from different parts of the region. Another criterion for sampling was that the patients of a selected hospital must reside throughout the respective study region.

The selected hospitals provided the medical records of patients with breast cancer diagnosed during 1999–2008. One month was randomly selected for each year for each hospital, and all pathologically diagnosed inpatient cases for that month were reviewed. January and February were excluded from the random selection to decrease any confounding effects of the Chinese New Year holiday (the longest holiday period of the year). If the number of qualifying cases was less than 50 in a selected month, the cases from the 1 month immediately before and the 1 month immediately after the selected month would be included until the total number in that year reached 50. If the number of inpatients in the selected month exceeded 50, all cases were reviewed.

A designed case report form was used to collect information on the enrolled patients, including general information, demographic characteristics, risk factors for breast cancer, results of clinical breast examination, diagnostic imaging, treatment process, and pathologic characteristics. All information was collected during a review of medical charts. The risk factors recorded were age at menarche, age at menopause, age at first delivery, number of live births, breastfeeding history, family history of breast cancer (ie, having ≥1 first-degree relative with a diagnosis of breast cancer), body mass index (BMI), use of oral contraceptives, and histories of regular smoking and regular alcohol drinking. The details of the data collection process have been described previously.^7^

### Representative data on selected factors of the general population

To assess the effects of selected risk factors on breast cancer among Chinese women, nationally representative population data were selected from national behavioral survey reports and the published literature and used as control data. Frequencies of behavior-related factors, including BMI, smoking, and alcohol drinking, were collected from Chinese national surveys of women in the general population. Due to the lack of national survey data on reproductive factors in the Chinese general population, we attempted to pool the reported exposure frequencies of selected factors among women without breast cancer from studies of risk factors for breast cancer in Chinese women. To ensure the representativeness of pooled controls, we used the following inclusion criteria. First, the recruitment periods of published studies had to coincide with that of the present study. Second, to ensure that the included controls would encompass the above-mentioned 7 geographic regions of China, studies conducted in the provinces and regions of the present study were preferentially selected. If there were no high-quality studies from 1 of the above provinces, data from studies conducted in neighboring provinces were collected. Finally, if multiple papers reported the same or overlapping data, the largest or most recent publication was selected.

We searched for studies published between 1995 and 2011 on the relationship between selected risk factors and breast cancer among Chinese women, using combinations of the following keywords: “breast cancer,” “risk factors,” and “Chinese women.” The databases searched were PubMed, Ovid, the Chinese Biomedical Literature Analysis and Retrieval System, and the Chinese medical databases, namely, National Knowledge Infrastructure and WanFang. All the subjects of the selected studies had to be recruited in 1995 or later. All included studies reported frequencies of cases and controls for the selected risk factors.

### Statistical analysis

The *t* test and χ^2^ test were used to compare the distributions of selected risk factors for breast cancer, stratified by regional economic development level (as determined by regional gross domestic product levels reported by Wang et al in 2012).^9^ We also compared women by menopausal status. After stratification by menopausal status, the trend χ^2^ test was used to identify trends in selected risk factors among women born in 4 birth periods: before 1950, 1950–1959, 1960–1969, and 1970 or later. To estimate the effect of selected risk factors for breast cancer among Chinese women, crude odds ratios (ORs) and their 95% CIs were calculated based on the frequencies of cases included in the retrospective studies and controls reported in included articles.

All *P* values were 2-tailed. SPSS 17.0 was used to perform χ^2^ tests, *t* tests, and trend χ^2^ tests and to calculate crude ORs and 95% CIs.

## RESULTS

A total of 4211 cases were identified during the 10-year period 1999–2008. Mean (SD) age at diagnosis was 48.7 (10.5) years.^7^ Because the rates of missing data for some risk factors (age at menarche, age at first delivery, and oral contraceptive use) were greater than 50%, we did not include those factors in the final analysis. For the included risk factors, the distributions for age at diagnosis and menopausal status were comparable between women with and without missing data.

### Distribution of risk factors for breast cancer among regions with different economic levels in China

The 7 regions were divided into less developed (middle, northwest, and southwest) and more developed (northeast, north, south, and east) areas (Table 1). There were 1528 cases recruited from the less developed regions (average [SD] age 48.7 [10.6] years) and 2683 cases (average [SD] age 48.6 [10.4] years) from the more developed regions. Because the proportions of cases with missing data for BMI, smoking, and alcohol drinking varied greatly by region, we only analyzed disparities in the distributions of number of live births, age at menopause, history of breastfeeding, and family history of breast cancer. As compared with those in less developed areas, cases in the more developed areas were more likely to be nulliparous and to have less childbirths (*P* < 0.05). Age at menopause, history of breastfeeding, and family history of breast cancer did not vary significantly by regional development level.

**Table 1.** Distribution of breast cancer risk factors by regional level of development

Variables	All cases	Level of development		Statistics (*P*)
	
	*n* (%) *N* = 4211	Less developed^a^ *n* (%) *N* = 1528	More developed^a^ *n* (%) *N* = 2683	
Age at diagnosis (y), mean ± SD	48.7 ± 10.5	48.7 ± 10.6	48.6 ± 10.4	0.58 (0.56)^b^
Age at menopause (y)				
Mean ± SD	49.1 ± 4.0	49.2 ± 3.4	49.1 ± 4.2	−0.97 (0.33)^b^
<50	688 (16.3)	275 (18.0)	413 (15.4)	2.13 (0.14)^c^
≥50	874 (20.8)	333 (21.8)	541 (20.2)	
Premenopausal	2649 (62.9)	920 (60.2)	1729 (64.4)	
Number of live births				
0	99 (2.4)	20 (1.3)	79 (2.9)	11.72 (0.003)^c^
1–2	3117 (74.0)	1107 (72.4)	2010 (74.9)	
≥3	731 (17.4)	251 (16.4)	480 (17.9)	
Missing	264 (6.3)	150 (9.8)	114 (4.2)	
History of breast feeding				
No	257 (6.1)	79 (5.2)	178 (6.6)	3.16 (0.08)^c^
Yes	2414 (57.3)	877 (57.4)	1537 (57.3)	
Unknown	1540 (36.6)	572 (37.4)	968 (36.1)	
Family history of breast cancer				
No	3984 (94.6)	1458 (95.4)	2526 (94.1)	0.21 (0.65)^c^
Yes	144 (3.4)	46 (3.0)	98 (3.7)	
Unknown	83 (2.0)	24 (1.6)	59 (2.2)	
Body mass index (kg/m^2^)				
Mean ± SD	23.4 ± 3.3	22.1 ± 2.4	23.7 ± 3.4	—^d^
<25	2360 (56.0)	698 (45.7)	1662 (61.9)	
≥25	921 (21.9)	84 (5.5)	837 (31.2)	
Missing	930 (22.1)	746 (48.8)	184 (6.9)	
Smoking history				
No	2468 (58.6)	1393 (91.2)	1075 (40.1)	—^d^
Yes	59 (1.4)	49 (3.2)	10 (0.4)	
Unknown	1684 (40.0)	86 (5.6)	1598 (59.6)	
History of alcohol drinking				
No	2427 (57.6)	1358 (88.9)	1069 (39.8)	—^d^
Yes	95 (2.3)	82 (5.4)	13 (0.5)	
Unknown	1689 (40.1)	88 (5.8)	1601 (59.7)	

^a^Less developed regions: middle, northwest, and southwest China; More developed regions: northeast, north, south, and east China.

^b^*t* test.

^c^Pearson χ^2^ test.

^d^Differences in distributions between regions were not compared due to large differences in the proportions of missing values.

### Distribution of risk factors for breast cancer in pre- and postmenopausal women in China

Among the breast cancer cases, 2649 were premenopausal (average [SD] age 43.1 [7.2] years) and 1562 were postmenopausal (average age 58.2 [7.9] years). As compared with premenopausal cases, postmenopausal cases had more live births and were more likely to be overweight and to have breastfed (*P* < 0.05; Table 2). There were no significant differences between pre- and postmenopausal cases in relation to history of smoking or alcohol drinking or family history of breast cancer.

**Table 2.** Distribution of breast cancer risk factors by menopausal status and birth period

Variables	Premenopause (*n*, %)					Postmenopause (*n*, %)			Pre- vs. Post-
		
	<1950	1950–	1960–	1970–	Total	<1950	1950–	Total	Statistics (*P* value)
Age at diagnosis (y), mean ± SD	58.4 ± 7.3	47.4 ± 3.3	40.5 ± 3.6	32.1 ± 3.6	43.1 ± 7.2	62.4 ± 6.7	51.4 ± 3.9	58.2 ± 7.9	26.32 (<0.001)^d^
Body mass index (kg/m^2^)									
<25	44 (49.4)	532 (68.3)	737 (79.8)	230 (88.1)	1543 (75.2)	457 (62.5)	360 (72.4)	817 (66.5)	28.33 (<0.001)^c^
≥25	45 (50.6)	247 (31.7)	187 (20.2)	31 (11.9)	510 (24.8)	274 (37.5)	137 (27.6)	411 (33.5)	
Statistics (*P* value)	81.76 (<0.001)^a^					13.07 (<0.001)^c^			
Number of live births									
0	7 (6.1)	20 (2.1)	14 (1.3)	29 (9.5)	70 (2.8)	16 (1.8)	13 (2.3)	29 (2.0)	483.89 (<0.001)^c^
1–2	65 (56.5)	848 (87.2)	1019 (94.9)	268 (87.6)	2200 (89.2)	429 (47.0)	488 (85.9)	917 (62.0)	
≥3	43 (37.4)	104 (10.7)	41 (3.8)	9 (2.9)	197 (8.0)	467 (51.2)	67 (11.8)	534 (36.1)	
Statistics (*P* value)	113.05 (<0.001)^a^					236.56 (<0.001)^c^			
History of breastfeeding									
No	8 (9.5)	57 (8.6)	69 (9.8)	47 (22.7)	181 (10.9)	41 (6.4)	35 (9.5)	76 (7.5)	8.29 (0.004)^c^
Yes	76 (90.5)	609 (91.4)	634 (90.2)	160 (77.3)	1479 (89.1)	602 (93.6)	333 (90.5)	935 (92.5)	
Statistics (*P* value)	18.99 (<0.001)^a^					3.31 (0.07)^c^			
Smoking history									
No	61 (98.4)	591 (98.0)	655 (97.2)	220 (98.2)	1527 (97.7)	559 (98.2)	382 (96.7)	941 (97.6)	0.02 (0.89)^c^
Yes	1 (1.6)	12 (2.0)	19 (2.8)	4 (1.8)	36 (2.3)	10 (1.8)	13 (3.3)	23 (2.4)	
Statistics (*P* value)	1.13 (0.75)^b^					2.36 (0.13)^c^			
History of alcohol drinking									
No	60 (96.8)	580 (96.5)	645 (95.7)	217 (96.9)	1502 (96.2)	552 (97.2)	373 (94.9)	925 (96.3)	0.002 (0.97)^c^
Yes	2 (3.2)	21 (3.5)	29 (4.3)	7 (3.1)	59 (3.8)	16 (2.8)	20 (5.1)	36 (2.4)	
Statistics (*P* value)	0.79 (0.86)^b^					3.33 (0.07)^c^			
Family history of breast cancer									
No	123 (94.6)	987 (97.2)	1065 (95.9)	320 (96.1)	2495 (96.4)	931 (97.8)	558 (95.1)	1489 (96.8)	0.42 (0.52)^a^
Yes	7 (5.4)	28 (2.8)	46 (4.1)	13 (3.9)	94 (3.6)	21 (2.2)	29 (4.9)	50 (3.2)	
Statistics (*P* value)	4.61 (0.23)^b^					8.64 (0.003)^c^			
Age at menopause (Years)									
<50	—	—	—	—	—	340 (35.2)	348 (58.4)	688 (44.0)	—
≥50	—	—	—	—	—	626 (64.8)	248 (41.6)	874 (56.0)	
Statistics (*P* value)	—	—	—	—	—	80.45 (<0.001)^c^			

^a^Trend χ^2^ test.

^b^Fisher exact test.

^c^Pearson χ^2^ test.

^d^*t* test.

### Birth period trends in the distribution of risk factors for breast cancer

Among premenopausal cases, the proportion of overweight women declined from the pre-1950 to the post-1970 birth period (*P* for trend <0.001; Table 2). The number of live births also progressively decreased from the pre-1950 to the post-1970 birth period, while the proportion of women without a history of breastfeeding increased (*P* for trends <0.001). The distributions of smoking, alcohol drinking, and family history of breast cancer did not vary significantly among by birth period. Among postmenopausal cases, the numbers of cases in the birth periods 1960–1969 and post-1970 were too small, so we combined all women born after 1950 into 1 subgroup for analysis. As compared with cases born in 1950 or earlier, cases born after 1950 had lower proportions of overweight and late menopause but higher proportions of nulliparity and family history of breast cancer (*P* < 0.001).

### Association between selected risk factors and breast cancer among Chinese women

Exposure information on smoking, alcohol drinking, and BMI among the general population of Chinese women, as reported in the 1996 National Prevalence Survey of Smoking Patterns^10^ and 2002 China National Nutrition and Health Survey,^11^^,^^12^ were treated as representative information on controls. Because the most recent national survey of age at menopause among Chinese women was conducted in 1990, a multicenter cross-sectional survey from 2005 that included the northern, southern, western, eastern, and central areas of China was selected to provide control information.^13^ For the other reproductive risk factors (number of live births, history of breastfeeding, and family history of breast cancer), 20 qualified studies were included, and covered the 7 regions of China included in the present study^14^^–^^33^ (Table 3). For risk factors with a large proportion of missing values, we calculated crude ORs and 95% CIs but excluded data from regions for which more than 30% of data were missing.

**Table 3.** Characteristics of included studies of the associations of risk factors with breast cancer among Chinese women

First author (year)	Region	Geographic location	Period of recruitment	Study design	Source of controls^b^	Age range (years)		Average age of controls (years)	No. of controls	Breast cancer-related risk factors^e^

						Lower	Upper			
Yang 1996^10^	30 provinces	Most of the country	1996	National survey	C	18	≥70	—^c^	34 124	A
Ma 2005^11^	31 provinces	Most of the country	2002	National survey	C	18	≥70	—^c^	79 840	B
Ma 2005^12^	31 provinces	Most of the country	2002	National survey	C	18	≥85	—^c^	82 989	C
Nie 2011^13^	Wuhan, Zhejiang, Shanghai, Beijing, Tianjin, Nanjing, Guangdong	North, South, West, East, Middle	2005.9–2005.11	Cross-sectional	C	41	60	—^c^	1248	D
Long 2010^14^	Shanghai (SBCS)^a^	East	1996.8–1998.3/ 2002.4–2006.12	Case-control	C	25	64	52.8 ± 9.2	1915	G
Shu 2001^15^	Shanghai (SBCS)^a^	East	1996.8–1998.3	Case-control	C	25	64	47.3 ± 14.5	1556	E
Sanderson 2001^16^	Shanghai (SBCS)^a^	East	1996.8–1998.3	Case-control	C	25	64	47.4 ± 8.7	1495	F
Zhou 2011^17^	Jiangsu	East	2001.3–2009.5	Case-control	C	22	85	49.9 ± 12.5	1116	G
Liu 2011^18^	Jiangsu	East	2004.6–2007.12	Case-control	C	<40	≥60	—^c^	682	E, F
Zheng 2000^19^	Shandong	East	1997.6–1999.4	Case-control	H	20	80	49.1^d^	404	E, F, G
Zhang 2009^20^	Tianjin	North	2004.12–2005.8	Case-control	H	—	—	50.4 ± 9.2	390	F
Tao 2009^21^	Beijing	North	2006.11–2007.4	Case-control	H	35	60	45.8^d^	781	G
Zhang 2011^22^	Guangdong	South	2007.6–2008.8	Case-control	H	25	70	—^c^	438	E, F, G
Zeng 2010^23^	Shenzhen	South	2000–2008	Case-control	H	21	79	45.1^d^	232	E, F
Han 2004^24^	Hubei	Middle	2002.1–2003.2	Case-control	H	≤39	≥60	49.1 ± 11.4	426	E, G
Zhang 2009^25^	Jiangxi	Middle	2005.11–2008.12	Screening program	C	22	68	46.3 ± 11.2	3667	F, G
Jiang 2010^26^	Henan	Middle	2009	Case-control	C	35	69	49.7 ± 9.2	134	F
Li 2006^27^	Liaoning	Northeast	2000.3–2005.1	Case-control	C	—	—	52.2^d^	620	E
Shi 2010^28^	Liaoning	Northeast	2008.1–2009.7	Case-control	H (14.9%) & C (85.1%)	21	75	48.0 ± 8.6	590	G
Li 2006^29^	Sichuan	Southwest	2003.12–2004.9	Case-control	C	30	70	45.6 ± 9.4	154	G
Zhao 1998^30^	Sichuan	Southwest	1994.6–1997.6	Case-control	H	28	82	46.8 ± 9.4	265	E, F
Nie 2008^31^	Yunnan	Southwest	1997.1–2007.4	Case-control	C	<30	>70	43.9^d^	200	G
Liu 2011^32^	Qinghai	Northwest	2002.6–2010.10	Case-control	H	21	79	42.1 ± 26.2	148	F
Zhang 1998^33^	Lanzhou	Northwest	1995.3–1996.6	Case-control	H	30	>60	—^c^	102	E

^a^SBCS: Shanghai Breast Cancer Study.

^b^H: hospital-based; C: community-based.

^c^Study did not report average age of controls.

^d^Study did not report SD of age of controls.

^e^Breast cancer risk factors—A. smoking; B. alcohol drinking; C. body mass index; D. age at menopause; E. number of live births; F. history of breast feeding; G. family history of breast cancer.

Among Chinese women, overweight, late menopause, and a family history of breast cancer were associated with elevated disease risk (crude ORs = 1.17–1.82, 95% CIs did not include unity). However, there was no significant risk associated with nulliparity, absence of a history of breastfeeding, or history of smoking and drinking (Table 4).

**Table 4.** Associations between selected risk factors and breast cancer among Chinese women

Variable	China		

	Cases^a^	Controls^b^	Crude OR (95% CI)
Body mass index (kg/m^2^) ≥24	1163 (38.2)^c^	26 058 (34.6)^12^	1.17 (1.08, 1.26)
Age at menopause ≥45 years	956 (88.5)^d^	1070 (85.7)^13^	1.28 (1.00, 1.64)
Nulliparous	99 (2.5)	115 (2.4)^15^^,^^18^^,^^19^^,^^22^^–^^24^^,^^27^^,^^30^^,^^33^	1.03 (0.79, 1.35)
History of breastfeeding (No)	257 (9.6)	692 (8.9)^16^^,^^18^^–^^20^^,^^22^^,^^23^^,^^25^^,^^26^^,^^30^^,^^32^	1.09 (0.94, 1.27)
Smoking history (Yes)	52 (2.5)^e^	909 (2.6)^10^	0.96 (0.72, 1.27)
History of alcohol drinking (Yes)	89 (4.4)^f^	3734 (4.5)^11^	0.97 (0.78, 1.20)
Family history of breast cancer (Yes)	144 (3.5)	189 (2.0)^14^^,^^17^^,^^19^^,^^21^^,^^22^^,^^24^^,^^25^^,^^28^^,^^29^^,^^31^	1.82 (1.46, 2.26)

^a^Reported in present retrospective study.

^b^Reported in previously published study.

^c^Areas with fewer than 20% missing values were included, namely, central, northeast, north, south, and east China.

^d^Data for women aged 41–60 years.

^e^Areas with fewer than 20% missing values were included, namely, central, northwest, southwest, and east China.

^f^Areas with fewer than 20% missing values were included, namely, central, northwest, southwest, and east China.

## DISCUSSION

This is the first nationally representative epidemiologic study of breast cancer risk factors in China. We found that the distribution of risk factors among patients differed by region, birth period, and menopausal status. These disparities could be due to differences in economic status and changing domestic policies.

Studies in Western countries revealed that socioeconomic inequality may be associated with the distributions of some breast cancer risk factors, including reproductive control and habits such as smoking and drinking.^34^^–^^36^ In the current analysis, we found that cases from more developed areas had fewer average births and larger proportions of nulliparous women and women who had not breastfed. Comparison with the control data in the present study showed that, among cases in more developed regions, the proportions of nulliparous women and women who had not breastfed were 2.41 and 3.55 times, respectively, those of the general population (nulliparity: OR = 2.41, 95% CI: 1.22–4.78; no history of breastfeeding: OR = 3.55, 95% CI: 2.98–4.22). These findings indicate that the disparities in the distributions of childbirth and breastfeeding among cases may be due to the characteristics of the general population.

One explanation for our findings is that, as in many other low-income countries with increasing economic prosperity, urbanization and modernization in more developed areas of China lead local women to adopt different lifestyles, including reproductive control. Another possible explanatory factor is that the 1-child policy in China is much more strictly enforced for families in more developed areas, which have largely urban populations.^37^ The 2001 National Family Planning and Reproductive Health Survey reported that the total fertility rate was 1.3 in urban areas and slightly less than 2.0 in rural areas.^37^

According to a current report on the Chinese Cancer Registry, the incidences of breast cancer in economically developed provinces were higher than those in developing areas. Thus, it is necessary to assess the population attributable risks of these reproductive factors for Chinese women, as this may help in understanding regional disparities in breast cancer incidence and devising proper prevention strategies in China.

During the past half-century, China has experienced dramatic social changes and rapid economic development. We therefore explored birth period trends in breast cancer risk factors. Because risk factors differ between pre- and postmenopausal women, we conducted the analysis of birth period after stratifying by menopausal status. The analysis identified several generational trends in breast cancer risk factors.

First, number of live births decreased significantly with time both in premenopausal and postmenopausal cases. This is probably due in large part to the 1-child policy that began in the 1970s. Second, the rate of breastfeeding declined among premenopausal cases, which may be the result of reform and liberalization policies that China adopted in the 1980s. Chinese women increasingly work outside the home and exert more reproductive control. The increase in breast cancer incidence during the past 2 decades indicates that the population attributable risks of these factors should be assessed, especially among younger women. Moreover, in conjunction with the decrease in child births, Chinese women might increasingly rely on oral contraceptives and induced abortions, both of which are suspected risk factors for breast cancer.^38^^,^^39^ Thus, their effects on breast cancer risk among Chinese women merit future study.

Among postmenopausal cases, we found age at menopause was earlier among women born after 1950 than among those born in 1950 or earlier. Earlier age at menopause may be related to the decline in average age at menarche among Chinese females. A previous study revealed that age at menarche among Chinese females declined to 14.6 years in 1957–1961 birth cohorts from 15.7 years in 1927–1940 birth cohorts.^40^ This may be due to improvements in childhood nutritional status during that period. Our findings appear to be inconsistent with the increase in breast cancer incidence in China. However, among East Asian women breast cancer was more likely to be diagnosed before menopause, and about two-thirds of the cases in our study were premenopausal, so the contribution of late menopause to overall breast cancer incidence in China may be small.^2^

We found that BMI decreased with time both in premenopausal and postmenopausal cases, despite the fact that BMI has increased among the general population in recent decades.^12^ This could be due to the decline in average age at diagnosis from the pre-1950 subgroup to the post-1970 subgroup in this cross-sectional study. Studies have shown that serum lipid and low-density lipoprotein levels, which are positively associated with BMI, increase with age among women.^41^

In the present study, BMI, age at menopause, and family history of breast cancer were associated with breast cancer risk among Chinese women. Similar findings were reported for Japanese women^42^^–^^44^ and American white women^45^ in large-scale population-based cohort studies (Japanese women: RRs = 1.61–2.67, 95% CIs did not include unity; American white women: HRs = 1.31–1.41, 95% CIs did not include unity). We found no associations between other selected factors and breast cancer among Chinese women. However, significant associations were reported among both Japanese women and American white women. However, the strength of these associations was low, as the relative risks ranged between 0.8 and 2.0.^43^^,^^45^^–^^49^

Our results suggest that the magnitude of the effects of risk factors may contribute slightly to differences in breast cancer incidence among countries. It was reported that disparities in exposure to risk factors may partly explain variation in breast cancer incidence among countries.^2^ Such exposures could be influenced by traditional culture and the extent of modernization. Genetic background may also explain disparities in exposures. Studies showed that women of Asian ancestry had lower proportions of mutation alleles of BRCA1 and DNA repair genes, which are associated with breast cancer susceptibility.^2^^,^^50^ Our results indicate that, among Chinese women, BMI, age at menopause, and family history of breast cancer should be given priority as environmental factors in the assessment of breast cancer risk. These factors differ from those used in the Gail model, which was developed for use among white women.^51^

About 40% of subjects had missing data on smoking and alcohol drinking. There was no significant difference in age between cases with and without information on either smoking or alcohol drinking (smoking: *t* = 1.44, *P* > 0.05; alcohol drinking: *t* = 1.55, *P* > 0.05). Similar results were observed for menopausal status (smoking: χ^2^ = 3.01, *P* > 0.05; alcohol drinking: χ^2^ = 2.76, *P* > 0.05). In addition, because exposures to the 2 factors were extremely low among Chinese women,^10^^,^^11^ their distributions are not likely to substantially differ between cases with and without missing information. Thus, the impact of missing data on the estimated effects of smoking and alcohol drinking is expected to be limited.

We verified the ages of controls, as age is the most important confounder. In present study, the age range of cases was 21 to 85 years, and average age was 48.7 years. For cultural reasons, the exposure of Chinese women to smoking and alcohol drinking has been very low.^10^^,^^11^ Therefore, age distribution might not greatly influence the estimated risks of these 2 factors. Family history of breast cancer is not believed to be associated with age. Although we detected disparities in distribution among birth periods in postmenopausal patients, this may have been a chance finding. When we divided patients into 10-year age groups, the disparity disappeared (χ^2^ = 5.97, *P* > 0.05). Thus, differences in age distribution are not likely to influence the estimated effect of family history of breast cancer.

With regard to age at menopause, the age range of controls was 41 to 60 years; 66% of cases in the retrospective study were in this age group. Due to the potential confounding effects of age, we recruited patients aged 41 to 60 years and calculated ORs. This group of patients was evenly distributed among the 7 regions (χ^2^ = 11.50, *P* > 0.05); thus, our results are likely to be reliable.

As for BMI, the proportion of controls younger than 45 years was about 1.4 times that of cases in our retrospective study. Because overweight is more likely among perimenopausal women, we conducted an analysis stratified by age. According to data from the national survey,^13^ the subjects were divided into 3 groups: 44 years or younger, 45 to 59 years, and 60 years or older. The effects of overweight on breast cancer were similar among these age groups (≤44 years: OR = 1.52, 95% CI 1.34–1.72; 45–59 years: OR = 1.59, 95% CI 1.43–1.77; ≥60 years: OR = 2.58, 95% CI 2.14–3.12). Use of a pooled odds ratio was thus deemed acceptable.

With regard to number of live births and breastfeeding, most previous studies were case-control studies of breast cancer. In previous research, cases were consecutively recruited from hospitals or randomly selected from communities, so the age distribution of those studies might be similar to that of the cases in the present study. Because most earlier studies matched controls and original cases by age, the age distribution of those studies probably does not vary greatly from that of the cases in the present study. As shown in Table 3, the age ranges of controls for each factor were similar to that of the cases in the present study. The average age of controls in most studies is close (±5 years) to that of the patients in the present study. In a study of a large sample from a screening program,^38^ age range and average age were similar to that of the present patients, so the impact of age on the associations of childbirth and breastfeeding history with breast cancer is likely to be slight.

This study has 2 limitations. First, it was hospital-based rather than community-based, so the cases may not be entirely representative of Chinese women with breast cancer. However, population-based disease surveillance systems are deficient in China, as the population is large and spread over a vast area. There is as yet no large-scale, geographically representative study of breast cancer risk factors among the general population. Second, we were unable to control for all potential confounders in the association of risk factors with breast cancer.

### Conclusion

As compared with cases from less developed regions, those from more developed regions were significantly more likely to be nulliparous, had fewer childbirths, and were less likely to have breastfed. These reproductive factors became more common in later birth periods. Because breast cancer incidence has recently increased and is higher in more developed regions, it will be necessary to evaluate the population attributable risks of these factors among Chinese women, especially in more developed areas and among young women. Our findings indicate that primary prevention strategies, including health education and policy modification, might prove useful. In addition, overweight, late menopause, and a family history of breast cancer were associated with elevated risk of breast cancer among Chinese women. Future studies should carefully evaluate the effects of BMI, age at menopause, and family history of breast cancer as predictive factors in breast cancer risk assessment for Chinese women.
